# Integrated management of groundwater quantity, physicochemical properties, and microbial quality in West Nile delta using a new MATLAB code and geographic information system mapping

**DOI:** 10.1038/s41598-024-57036-8

**Published:** 2024-04-02

**Authors:** Mohamed Shehata, Samir M. Zaid, Soha T. Al-Goul, Ashwag Shami, Khalid M. Al Syaad, Ahmed Ezzat Ahmed, Yasser S. Mostafa, Diana A. Al-Quwaie, Mada F. Ashkan, Fatimah S. Alqahtani, Yusuf A. Hassan, Taha F. Taha, Khaled A. El-Tarabily, Synan F. AbuQamar

**Affiliations:** 1 Geology Department, Faculty of Science, Zagazig University, Zagazig, 44511 Egypt; 2 Department of Chemistry, College of Science and Arts, King Abdulaziz University, Rabigh, 21911 Saudi Arabia; 3 Department of Biology, College of Science, Princess Nourah bint Abdulrahman University, Riyadh, 11671 Saudi Arabia; 4 Biology Department, College of Science, King Khalid University, Abha, 61413 Saudi Arabia; 5 Prince Sultan Bin Abdelaziz for Environmental Research and Natural Resources Sustainability Center, King Khalid University, Abha, 61421 Saudi Arabia; 6 Biological Sciences Department, College of Science and Arts, King Abdulaziz University, Rabigh, 21911 Saudi Arabia; 7 Department of Biology, Faculty of Sciences, University of Bisha, Bisha, 61922 Saudi Arabia; 8 Biochemistry Department, Faculty of Agriculture, Zagazig University, Zagazig, 44511 Egypt; 9 Department of Biology, College of Science, United Arab Emirates University, Al Ain, 15551 United Arab Emirates

**Keywords:** Bacterial count, Drawdown simulation, GIS, Groundwater, Physicochemical quality, Quaternary aquifer system, Salinity maps, Freshwater ecology, Environmental impact

## Abstract

Groundwater is an excellent alternative to freshwater for drinking, irrigation, and developing arid regions. Agricultural, commercial, industrial, residential, and municipal activities may affect groundwater quantity and quality. Therefore, we aimed to use advanced methods/techniques to monitor the piezometric levels and collect groundwater samples to test their physicochemical and biological characteristics. Our results using software programs showed two main types of groundwater: the most prevalent was the Na–Cl type, which accounts for 94% of the groundwater samples, whereas the Mg–Cl type was found in 6% of samples only. In general, the hydraulic gradient values, ranging from medium to low, could be attributed to the slow movement of groundwater. Salinity distribution in groundwater maps varied between 238 and 1350 mg L^−1^. Although lower salinity values were observed in northwestern wells, higher values were recorded in southern ones. The collected seventeen water samples exhibited brackish characteristics and were subjected to microbial growth monitoring. Sample WD12 had the lowest total bacterial count (TBC) of 4.8 ± 0.9 colony forming unit (CFU mg L^−1^), while WD14 had the highest TBC (7.5 ± 0.5 CFU mg L^−1^). None of the tested water samples, however, contained pathogenic microorganisms. In conclusion, the current simulation models for groundwater drawdown of the Quaternary aquifer system predict a considerable drawdown of water levels over the next 10, 20, and 30 years with the continuous development of the region.

## Introduction

Sustainable freshwater management is vital for the environment and human health. Climate change, industry, urbanization, population growth, and chemical pollution can cause water problems worldwide, particularly in arid and semi-arid regions^1–3^. Water-related issues have afflicted the Middle Eastern, Central Asian, and certain Southeast Asian states. It has been projected that these battles will culminate in disputes over shared water supplies in these locations. Many countries have adopted eco-friendly water management techniques and explored alternative water supply sources to mitigate the adverse effects of the water shortage^2–4^. Some methods for water management and combating freshwater problems include conservation and reuse of water and purification of brackish groundwater and saltwater^1–4^. Therefore, countries strive to find alternative water supplies to supplement freshwater sources i.e., groundwater. The latter has been considered the second largest source of freshwater after the Nile River.

Freshwater represents only 2.5% of the Earth's water only, while 30.1% of the available freshwater is groundwater^5^. Freshwater sources must be increased to meet future challenges (growth in population and pollution of water resources) and economic growth^6–8^. About 10–30% of the freshwater resources are consumed by human, mainly in industry and agriculture^9^, which lower the local water supply^10,11^. Intensive human activities can be added to the stress on the environment and ecosystems, deteriorating soil and water resources and severely restricting sustainability^12,13^. Chemical substances and microorganisms in groundwater and surface water also cause pollution^13–15^. Chemical compounds have a considerable influence on water suitability for human consumption, and industrial and agricultural use. Although several researchers have undertaken extensive investigations on various topics addressing water contamination, it is clear that mature and scientifically sound procedures are lacking in several areas^16^. This can be attributed to the difficulty in defining the critical criteria for measuring water chemistry along with the effects of processes that regulate water quality^17^.

Groundwater is a vital freshwater resource for drinking, irrigation, and industry that has a visible economic role. Groundwater is essential in dry lands as there are no available water resources. About 70% of groundwater worldwide is used for agriculture, while 3.2% is used in industry^8,18,19^. Aquifers differ locally and regionally, with variable depths, recharge, and media of the water body^20^. Most aquifers comprise unconsolidated or consolidated granular (sand and gravel) material or fissured limestone.

Groundwater is divided into two categories: Renewable and non-renewable groundwater resources. Groundwater is an essential and accessible, even during dry seasons. As a result of the rapid progress in industry and the rise in population, global urban groundwater consumption is rising^21^. Groundwater is of high quality because it is filtrated through rocks and soil. Because not all soils are efficient filters, many pathogens in human waste might flow through the soil filter and pollute the groundwater^22^. The susceptibility of groundwater sources to contamination varies geographically and temporally under various degrees of source degradation^23^. Therefore, developing a geographical groundwater quality database is essential to monitor and regulate its usage.

Microbiological analysis of groundwater may significantly contribute to identify water-borne pathogens that harm our health, and may potentially help find appropriate remedies, including decontamination and water treatment^24^. In addition to resolving water quality concerns, a microbiological examination of groundwater is extremely important for comprehending the geochemical behavior of an aquifer. Inorganic mechanisms impacting leaching and transportation of hazardous substances into deeper subsurface horizons are characterized by microbiological analysis^25^.

Geochemical modeling^26^ requires an accurate appraisal of microorganisms to address the fate of contaminants. Consequently, water quality index (WQI) is a mathematical technique that significantly simplifies the data associated with water quality while delivering a single classification value that describes the quality status or contamination level of bodies of water^27^.

Considering that groundwater quality is a continuous field, a map for groundwater quality is a valuable tool for managing this resource to encourage optimal utilization and maintain public health^28^. Due to the high cost and limited time for sampling groundwater at every place, predicting the groundwater quality at other locations based on measured values may provide a relatively reasonable solution. In this situation, geographic information system (GIS) approaches, in conjunction with geostatistics, may be used to forecast groundwater quality^29^. GIS-based mapping of Ain Sefra, Algeria, examined the adequacy of groundwater quality. Forty-three samples of groundwater were collected in March 2022, and various physicochemical limits were analyzed within Gibbs and Piper diagrams. Likewise, the ArcGIS tool performed spatial distribution maps of twelve main water quality parameters using the inverse distance weighted (IDW) interpolation method. The WQI has been calculated considering the Algerian drinking water quality standards to know the suitability of water for human consumption^30^. Derdour et al.^31^ also assessed water quality in arid regions. They monitored the status of groundwater quality based on hydrochemical parameters using artificial intelligence approaches, such as irrigation water quality index (IWQI), and k-nearest neighbours (KNN) classifiers in Matlab's classification learner toolbox.

In the current study, we used GIS and geostatistical approaches to evaluate the groundwater quality in Sadat City, Menoufia Governorate, Egypt, to establish a geographic groundwater quality database for any ongoing/future monitoring/planning. We spatially investigated the physiochemical and microbiological quality of the groundwater, the change in the water level drawdown, and the salinity distribution of freshwater in the Quaternary aquifer. The novelty in our study was that the distribution of dissolved minerals in the groundwater was monitored using a new MATLAB code, while obtaining time series models for changing water levels after development and forecasting groundwater's physiochemical and microbial quality at unsampled places.

## Materials and methods

### Experimental study area

**Figure 1:**
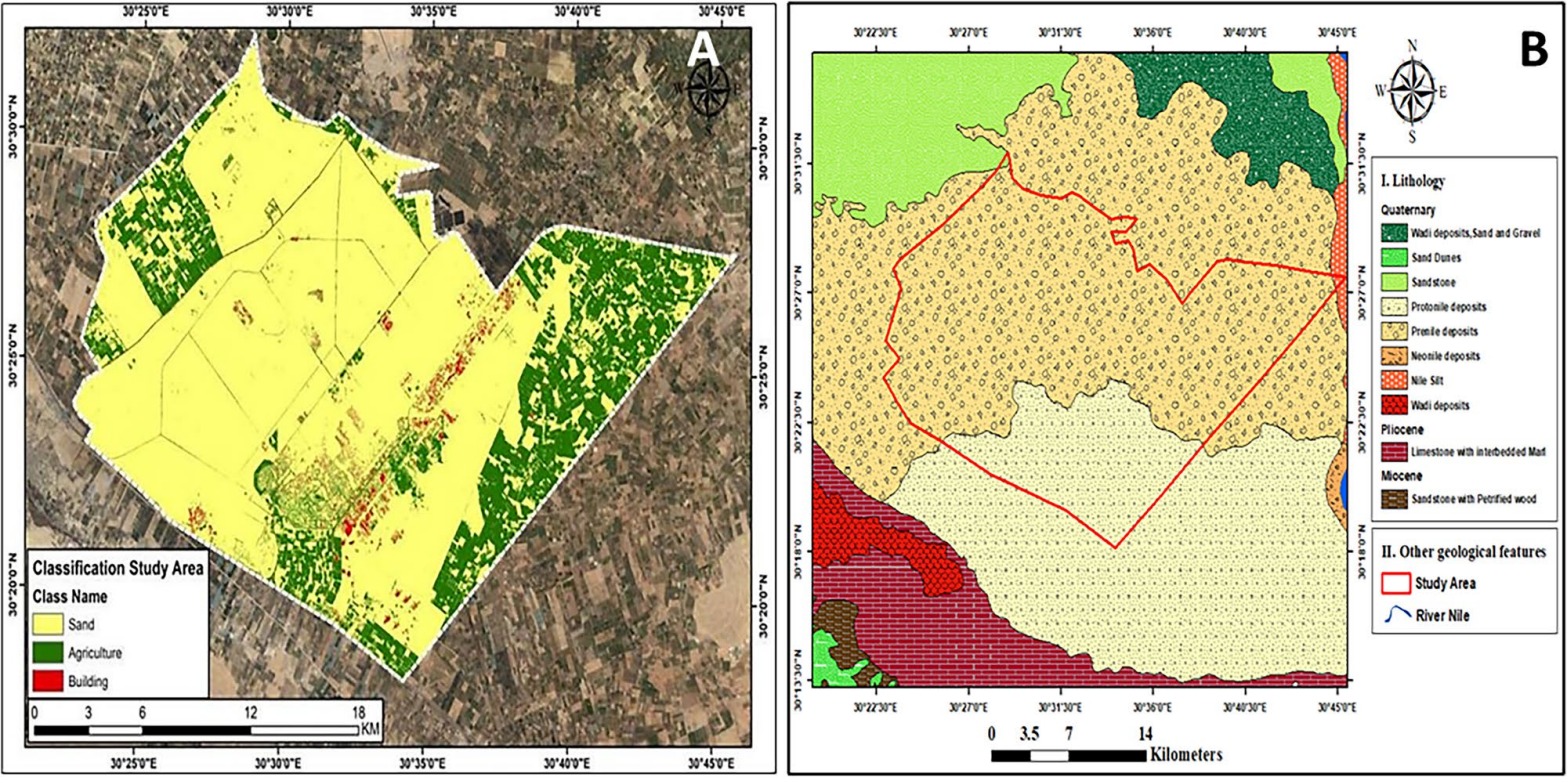
Location of the experimental study area. A map showing (**A**) the agricultural land distribution, (**B**) the geological map of Sadat City, Menoufia Governorate, Egypt. Figures were generated using ArcGIS 10.7 software (www.esri.com/en-us/arcgis/products/arcgis-desktop/resources).

The study area was carried out in Sadat City, Menoufia Governorate, Egypt (latitudes 30°26′ 30.36″ and 30°23′ 47.87″N and longitudes 30° 39′8.51″ and 30°26′ 43.4″E). This area is a significant agricultural region in the Western Nile Delta that consists of the green belt (16,380 hectares), and green landscapes (5040 hectares). The cultivated areas in the study area represents about 42% of the total area. This study area is considered for sustainable development and reclamation of land. The distribution of agricultural fields in the study area is shown in (Fig. 1A). In addition to planting economic crops (e.g., corn), the productivity of seasonal economic crops, such as onions, wheat, rice, beans, barley, lupine, clover sprouts, flax, strawberry, mango, and oranges, might be enhanced if water sources increased.

Concerning the geo-characteristics, the study area contains crystalline and sedimentary rocks, such as sandstone, carbonate rocks, or evaporites, belonging to the Neogene and the Quaternary periods. The Nile Delta, located east of the studied area, formed a tectonic depression, which subjected a thick series of argillaceous strata deposited in the Nile Delta to general subsidence. The northwestern desert was a positive area during much of the Tertiary period. During the Miocene period, sands were deposited in a fluvial environment. The thicknesses of sands ranged between 200 and 1000 m, increasing in the northward direction. During Pliocene times, lagoonal facies developed. Sedimentary rocks mainly occupy the studied area in the Upper Cretaceous, Tertiary, and Quaternary periods^32^ (Fig. 1B).

### Climate of the study area

In addition to 2021, climate data from 2009 to 2017 were collected for the current study. Data obtained from the meteorological stations include temperature, rainfall, atmospheric pressure, and humidity were also collected.

#### Temperature

The temperature values in the region range from high to moderate in the summer season (June, July and August), where the maximum and minimum temperature were 45.91 °C and 42.85 °C, respectively. In winter (December, January, and February), temperatures were 6.41 °C (the maximum) and 2.01 °C (the minimum). There was relative stability of annual temperature variations in the study area between 2011 and 2021 and very low-temperature fluctuations according to NASA/POWER CERES/MERRA2.

#### Rainfall

Data from the meteorological stations, that are well-distributed in the present study, showed the monthly rainfall quantity between January 2011–December 2021. The rainy season extends in this area from January to April; whereas the non-rainy season is from May to December. In general, there was an increase in the precipitation quantities during the rainy season between 2011 and 2021. According to the Native Resolution Monthly and Annual by NASA/POWER CERES/MERRA2, the climate is arid to semi-arid, with total rainfall of 0.14–0.44 mm year^−1^, indicating a low rainfall rate in the study area. The highest rainfall was recorded in April 2020; however, the region suffered in months of June–September with scarcity of rainfall of the same year. The years of 2011, 2012, 2013, 2016, 2018, and 2019 were the wettest.

#### Humidity

The relative humidity was mostly constant, ranging between 53.31–59.19%. This is likely due to the continuous evaporation of water bodies in the study area. The highest air humidity value was recorded in December 2011, and the lowest was in May 2019 (NASA/POWER CERES/MERRA2).

### Regional hydrogeology

Four geomorphological units comprise the western portion of the Nile Delta: Young and ancient sedimentary plains, fanglomerates, and dunes. Young plains dominate the farmed regions, flanking the river channel and branches of the Nile. Most of the Nile Delta comprises these plains, split by irrigation ditches and drains, gradually sloping from south to north and on both flanks towards the river channel. The elevation varies from around 18 m above sea level (m asl) in the south to approximately 4 m asl in the north, with a mild slope of approximately 1 m 10 km^−1^. The ancient sedimentary plains are located south of the new ones and ranged between 20 and 60 m in height. These plains encompass most of the freshly recovered land on the western margins of the Nile Delta. Fanglomerates reflect wadi wash carried in by drainage lines and deposited in shallow depressions before entering the Nile. On both sides of the Nile Delta, there were well-developed transverse dunes of several hundred-meter-long sand bars oriented primarily NNW–SSE^33^.

**Figure 2:**
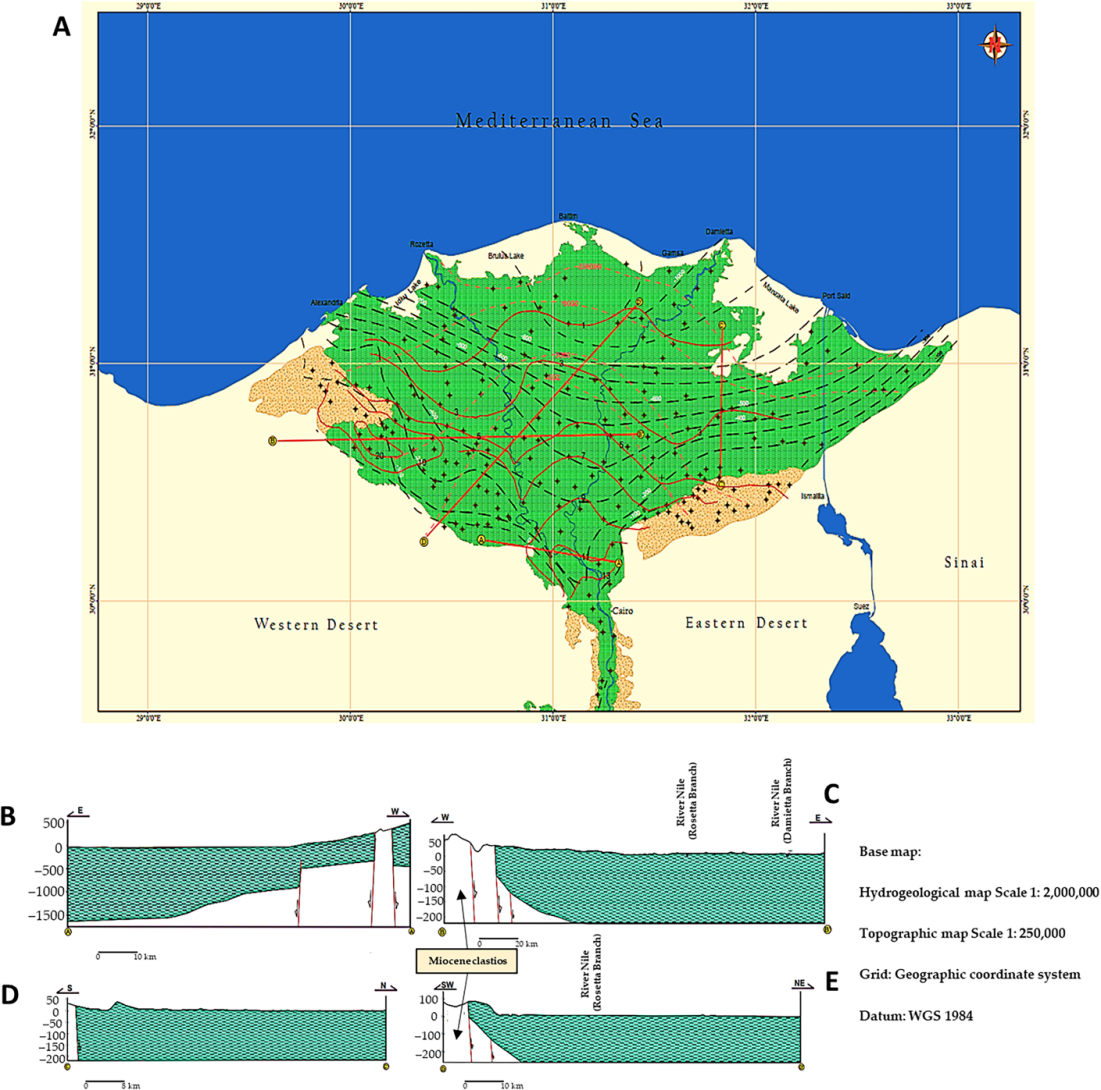
Hydrogeological mapping of the study area. The hydrogeological (**A**) main map; and (**B-E**) cross-sections of Rosetta and Damietta branch regions of the River Nile, Egypt. Figures were generated using ArcGIS 10.7 software (www.esri.com/en-us/arcgis/products/arcgis-desktop/resources).

The Western Nile Delta surface is dominated by sedimentary outcrops aged from the Late Cretaceous to the Quaternary (Fig. 2)^34^. Concurrently, the underlying geologic sequence consists of Triassic, Jurassic, Cretaceous, Eocene, Oligocene, Miocene, Pliocene, and Quaternary rock groups. They are composed of three consecutive layers (Table S1): (1) The first layer, at a depth of 0–60 m, is made of rock fragments that are medium to fine sand, quartz, pale yellow, transparent, off-white, colorless, hard with clay, in part brown, soft, and flakey; (2) the second layer, at a depth of 60–160 m, is made of fine to medium sand, quartz, colorless, light grey, off-white, and hard; and (3) at 160–200 m depth, the third layer is fine to medium sand, quartz, colorless, light grey, off-white, complicated with shale, hard, grey, and sticky. This extensive sedimentary layer has a total depth of approximately 4000 m and lies unconformably on the basement complex. The western Nile Delta is classified using remote sensing using Landsat imagery. They statistically examined and categorized the lineaments (fractures and faults) according to their tendencies into three primary sets: N55°W–S55°E, N85°W–S85°E, and N75°E–W. The terrain is mildly undulating, with a few low hills in the west and primarily easterly and northerly slopes toward the Nile Delta and the Mediterranean Sea. Quaternary water formation in the study area is formed mainly of fluviatile-graded sand and gravel, including the lower Pliocene pyretic clay or sandy clay, which defines clay lenses and its base. The water table defines its upper limits. The Quaternary sediments show variable thicknesses^35^.

### Groundwater movement and flow direction

Concerning the recharge and discharge of groundwater in the flood plain of River Nile, the groundwater of the Quaternary Nile delta aquifer is continuously recharged by infiltration of surface water. This comes directly from the main canals or indirectly through excess irrigation water. Rainfall contribution to the recharge of the Nile Delta aquifer is minor; during high rainfall periods, wadi runoff occurs towards the playas. Infiltration of this runoff water may take place occasionally.

The groundwater discharge occurs through three mechanisms: (a) outflow into the drainage system in the Nile Delta floodplain, (b) outflow to other aquifers, and (c) groundwater extraction. The River Nile acts as a drain, and the main drainage canals contribute to the groundwater discharge of the Nile Delta aquifer.

**Figure 3:**
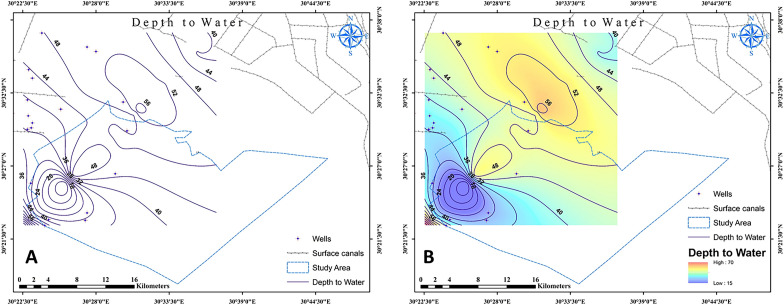
Quaternary aquifer system in the study area (September 2021). The (**A**) wells and (**B**) depth to water. The numbers in the figure expressed the groundwater depth in the study area. Figures were generated using ArcGIS 10.7 software (www.esri.com/en-us/arcgis/products/arcgis-desktop/resources).

Water depth measurements at groundwater monitoring wells throughout the study area and ground elevations at well sites were used to construct a study area's potentiometric surface map (Fig. 3). A Quaternary aquifer system potentiometric surface map suggests that the groundwater flow are directed through depth to water contour lines (Fig. 3). The elevation map were distinguished into three significant parts according to high, moderate, and low elevations. In the northern part of the map, the groundwater flow was observed to be in two directions: one to the southwest and the other to the east. In the middle part of the map, groundwater movement had a flow to the northeast. In the southern portion, the groundwater flow was generally towards the northeast and southeast directions.

### Distribution of water sources

**Figure 4:**
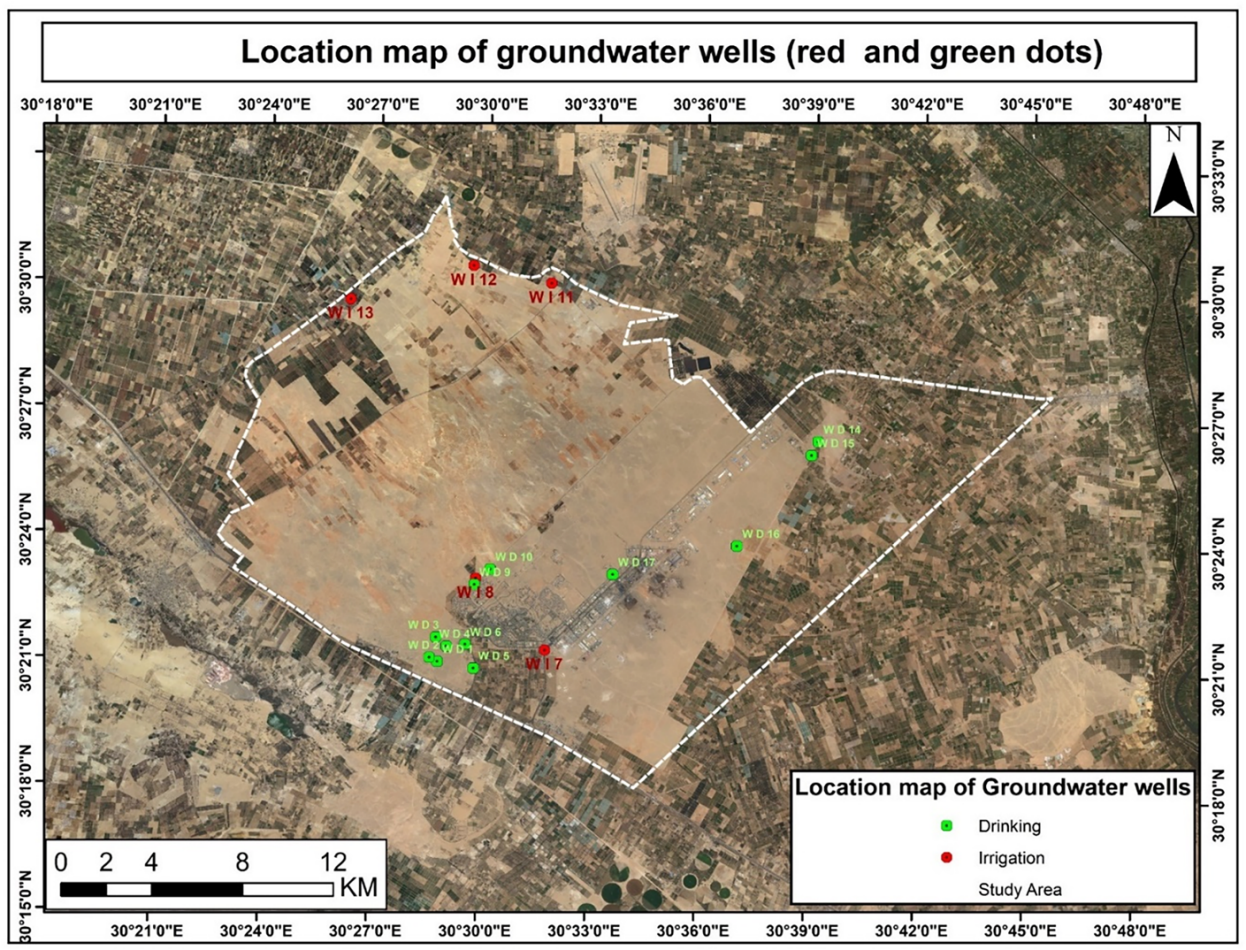
Location map of the groundwater’s well in the study area. Figure was generated using ArcGIS 10.7 software (www.esri.com/en-us/arcgis/products/arcgis-desktop/resources).

The water depth was measured using a meter scale. The pH and electrical conductivity (EC) were also measured using a pH meter (Jenway 3510, Cole Parmer Inc, United Kingdom) and an EC meter (HANNA Instruments HI9813-61 Portable EC, Smithfield, RI, USA) in the field. Seventeen samples from various water sources were collected and transferred into sterilized bottles to the laboratory for physicochemical and microbiological analyses for 24 h. Twelve samples were from drinking wells, and five were from irrigation wells. These samples were collected based on the well distribution of water sampling sites, with each variation in the sedimentary facies being represented by the collected water sample. The distribution of the various water sources was determined using GIS (Fig. 4).

### Physiocochemical analysis

Seventeen water samples in 1-L bottles were filtered through Whatman 41 filter paper to separate suspended solids; then, alkaline KI was added to prevent microbial contamination. The bottles were labeled and kept in a refrigerator at 4 °C for further analysis.

The following hydrochemical and physicochemical parameters were measured to test groundwater quality: Total dissolved solids (TDS), pH, EC, total alkalinity (TA), total hardness (TH), in addition to the ion and mineral concentrations. The TDS content was quickly determined using a TDS meter (HANNA Instruments HI9813-61 Portable TDS, Smithfield, RI, USA). The pH and EC of water samples were also determined^36^. Through titration with a bromo cresol green-methyl red indicator, TA was determined and evaluated as bicarbonate (HCO_3_^−^)^36^. TH was determined by titration with an EDTA-coupled Eriochrome Black-T indicator (Sigma-Aldrich Chemie GmbH, Taufkirchen, Germany). A flame photometer (Jenway PFP7, Chelmsford, Essex, United Kingdom) was used to determine sodium (Na), potassium (K), calcium (Ca) and magnesium (Mg) amounts, while Cl^−^ and sulfate (SO_4_^−2^) concentrations were determined by indirect UV detection^37^. Each analysis was performed three times using the mean values.

### Microbial counts

For each sample, five replicates were mixed of which a representative sample was collected. Representative water samples (10 mL) were mixed with 90 mL of sterilized peptone buffer and serially diluted up to 10^−7^. The microbial count was expressed for water samples as log colony forming unit (CFU) mL^−1^, as previously described^38^. The total viable count (TVC) was determined using plate count Agar (PCA; Lab M Limited, Lancashire, United Kingdom) after two days of incubation at 30 °C. On rose-Bengal chloramphenicol Agar (Lab M), the total yeast and mold count (TYMC) was determined after incubation for five days at 25 °C. The total number of coliforms was determined after 24 h of incubation at 37 °C on violet red bile (VRB) agar (Lab M). *Escherichia coli* were counted after 24 h of incubation at 37 °C on tryptone bile *(*TBX) agar (Lab M). On Chromocult® enterococci agar (Lab M), red colonies indicated the presence of *Enterococcus* spp.

The microbial load was served for physicochemical identification, which indicates that all counts in collected water samples contained coliform bacteria and *Streptococcus* spp. These isolates were molecularly identified using 16S RNA gene sequence by polymerase chain reaction (PCR). *Citrobacter freundii, Enterobacter cloacae, Enterobacter aerogenes, E. coli, Klebsiella pneumoniae, Streptococcus bovis,* and *S. equinus* were isolated. For algal isolation, single cells were selected and picked from the sample using a micropipette or glass capillary under microscopic observation. These cells were transferred to sterile algae culture agar (ACA, Oxoid, Thermo Fisher Scientific, Wilmington, DE, USA).

Based on Environmental Protection Agency (EPA) guidelines^39^, the quality of collected water samples should be in the range of pH = 6.0–9.0 (measured weekly), five-day biochemical oxygen demand (BOD5) ≤ 10 mg L^−1^ (measured weekly), Cl_2_ residual ≤ 1 mg L^−1^, turbidity ≤ 2 NTU (monitored daily), and fecal coliforms = 0/100 CFU (monitored daily). According to the Egyptian standards (ES), World Health Organization (WHO), and Canadian Council of Ministers of the Environment (CCME) standards for water quality for human and plant use, the chemical and microbial analysis are presented in Table 1.

**Table 1:** Standard water parameters according to guidelines provided by Egyptian standards (ES), World Health Organization (WHO), and the Canadian Council of Ministers of the Environment (CCME).

Parameters	Units	Methods	ES,458^78^	WHO^79^	CCME^80^
pH		Potentiometry (pH meter)	8.5	6.5–8.5	6.5–9
EC	μS cm^−1^	Conductivity probe	< 1200	2000	< 1800
TH	mg L^−1^	Complexometry by EDTA titration	500	500	
Ca		Argentometry (titration)	75	75	
Mg		Argentometry (titration)	50	50	
HCO_3_		Phenolphthalein and methyl orange indicators	< 500	< 250	
TA		Titration	500	500	
Na		Flame photometer analysis	200	200	500
K		Flame photometer analysis	10	10	< 150
Cl		Titration	200	250	< 10
SO_4_		Spectrophotometer	200	250	120
TDS		TDS probe	500	1000	
NH_3_		Titration	0.20	0.45	< 500
NO_3_		Spectrophotometer	11	10	1.37
PO_4_^–3^		Spectrophotometer	< 0.40	1	2.93
Total Fe		Spectrophotometer	0.3	0.3	
Mn		Spectrophotometer	0.1	0.1	0.3
Cu		Spectrophotometer	2	2	0.05
Zn		Spectrophotometer	0.5	3	0.004
CN		Spectrophotometer	< 0.05		0.05
Fecal coliform	CFU mg L^−1^	Plate count	0	0	0

*EC* electrical conductivity, *TH* total hardness, *Ca* calcium, *Mg* magnesium, *HCO*_*3*_ bicarbonate, *TA* total alkalinity, *Na* sodium, *K* potassium, *Cl* chloride, *SO*_*4*_ sulfate, *TDS* total dissolved solids, *NH*_*3*_ ammonia, *NO*_*3*_ nitrate, *PO*_*4*_^*−3*^ phosphate, *Fe* iron, *Mn* manganese, *Cu* copper, *Zn* zinc, *CN* cyanide, *EDTA* ethylenediaminetetraacetic acid, *CFU* colony forming units.

### Programming and database

#### GIS techniques

GIS is a tool used for reviewing and analyzing data for mapping purposes. Spatial Analyst and Model Builder were used to perform calculations and generate maps using ArcGIS version 10.7 software (Esri North Africa, Egypt; www.esri.com/en-us/arcgis/products/arcgis-desktop/resources). Building a model to generate several thematic layers was used as input data. Layers were analyzed to determine the occurrence of the distribution of topographic regions. These layers allow the identification of areas with high levels of vulnerability^39^. Other layers analyze the distribution of dissolved substances in groundwater. These layers can give the first overview of the availability of groundwater supplies in the local areas. The map shows sampling points that were imported into ArcGIS software, georeferenced and digitized. The point layer imported the sampling points of the different locations into GIS software. Each sample point was assigned another code and stored in the attribute table. Selected parameter data is linked to the sampling locations using the geodatabase creation function of ArcGIS 10.7 software. Using a geodatabase, we generated the spatial distribution maps of the chemical indices.

#### Interpolation analysis

Interpolation predicts the values of cells in a raster from a limited number of sample data points. It can predict unknown values of geographic point data, such as elevation, rainfall, chemical concentrations, and noise levels. The assumption that makes interpolation a viable option is that spatially distributed objects are spatially correlated. In other words, items that are close together tend to have similar characteristics. The present study uses two spatial interpolation methods: inverse distance weighted (IDW) and topo to raster for the chemical indices and digital elevation models (DEMs), respectively.

##### IDW

IDW is an interpolation method in which estimates are made based on values at nearby locations weighted only by distance from the interpolation location^40^. This method assumes that the variable being mapped decreases in the influence of distance from its sampled location; therefore, IDW can be used to map the distribution of the chemical indices.

##### Topo to raster

Topo to raster interpolates the elevation values for a raster while imposing constraints to ensure a connected drainage structure and a correct representation of ridges and streams from input contour data. This method has been designed to take advantage of the types of available input data and the known characteristics of elevation surfaces. It uses an iterative finite difference interpolation technique. It is optimized to have the computational efficiency of local interpolation methods (e.g., IDW) without losing the surface continuity of global interpolation methods (e.g., Kriging and Spline). It is a discretized thin plate spline technique^40,41^ for which the roughness penalty has been modified to allow the fitted DEM to follow abrupt changes in terrain, such as streams, ridges, and cliffs.

##### Generation of a fishnet grid of distribution with ArcGIS

Many environmental projects establish a design for the presence or abundance of maps in a fishnet (grid net). Depending on their abundance, we presented a grid using ArcGIS 10.7 software for the number of sightings of wells inside an area with points. We generated a 1 km × 1 km grid net that extends throughout the whole region of the development areas (16,380 and 5040 ha).

#### Monitoring groundwater levels using MATLAB

##### Developing time series models

Time series models are easy to construct and have high accuracy. Time series models represent the course of the groundwater table at a single point in three-dimensional (3-D) space. Groundwater levels were observed and factors (precipitation, evapotranspiration, and hydrological interventions) affecting the groundwater level were also incorporated. This was used to generate time series models of all groundwater levels. These models help in the quantification of the influence of factors/measures on the groundwater level, detection and quantification of the trends in the groundwater level, and filtering, lengthening, or filling up the short or messy groundwater-level observations.

##### MATLAB code

MATLAB coding was used to generate the first simulation of the drawdown process in the wells located on the Quaternary aquifer, which is the two areas of development in Sadat City as described above.

By using ArcGIS 10.7 software, the geodatabase functionality determined the sampling locations and the chemical index spatial distribution maps. IDW and topo to raster methods were used to show the chemical index distribution. These two methods interpolated the estimates that are dependent on at close-by locations only to the distance from the interpolation location.

Our methodology dealt with the groundwater quality, the change in the water level drawdown, and the freshwater distribution salinity in the Quaternary aquifer through spatial analysis^42^. In addition, the distribution of dissolved minerals in groundwater was monitored using a new MATLAB code to obtain time series models for changing water levels after development.

By harnessing the power of new MATLAB code, we developed accurate models for long-term (10, 20, 30 years) simulations of groundwater level drawdown in the Quaternary aquifer. These models incorporated critical factors like precipitation, evapotranspiration, and hydrological conditions. This would enable effective monitoring of drawdown trends, data completion, and trend analysis, ultimately supporting informed decision-making regarding water resource management in the development area.

### Statistical analysis

All experiments were performed in triplicates. All data are expressed as the mean ± standard deviation (SD). One-way Analysis of Variance (ANOVA) was used to statistically analyze the mean values, where *P* ≤ 0.05 was used to determine the significant difference. The least significant difference (LSD) test was used to statistically compare the means among water samples to enhance the ground water quality.

## Results

### Physiochemical properties of groundwater samples

The physiochemical qualities of groundwater data were analyzed (Table 2). Ten parameters were used to interpret the groundwater quality in the Quaternary aquifer area.

**Table 2:** Physicochemical analysis of groundwater samples in September 2021.

Sample	pH	EC	Concentration (mg L^−1^)							
			Ca^+2^	Mg^+2^	CO_3_^−2^	Na^+^	K^+^	Cl^−^	SO_4_^−2^	TDS
WD1	8.10 ± 0.1 ^c^	876 ± 1.1 f.	48 ± 0.2 ^e^	55.2 ± 0.2 ^b^	85 ± 0.2 ^c^	85 ± 0.8^d^	78 ± 0.5 ^d^	145 ± 0.3 ^e^	40 ± 0.1 ^e^	438 ± 1.1 ^e^
WD2	7.90 ± 0.1 ^e^	778 ± 0.9 ^g^	44 ± 0.3 ^e^	21.6 ± 0.8 ^d^	80 ± 0.1 ^c^	79 ± 0.2^d^	70 ± 0.1 ^d^	135 ± 0.9 ^e^	37 ± 0.2 ^e^	389 ± 1.0 ^ef^
WD3	7.95 ± 0.2 ^e^	862 ± 1.2 f.	44 ± 0.5 ^e^	24.0 ± 0.6 ^d^	82 ± 0.3 ^c^	84 ± 0.1^d^	76 ± 0.2 ^d^	138 ± 1.2 ^e^	39 ± 0.4 ^e^	431 ± 0.9 ^e^
WD4	8.13 ± 0.3 ^c^	1786 ± 0.8 ^c^	88 ± 0.8 ^bc^	48.0 ± 0.2 ^bc^	120 ± 0.4 ^b^	210 ± 0.9^b^	180 ± 0.3 ^b^	270 ± 1.1 ^c^	110 ± 0.5 ^c^	893 ± 1.2 ^c^
WD5	8.50 ± 0.5 ^a^	2700 ± 0.5 ^a^	104 ± 0.4 ^a^	64.8 ± 0.1 ^a^	160 ± 0.2 ^a^	350 ± 0.8^a^	290 ± 0.9 ^a^	410 ± 1.0 ^a^	250 ± 0.6 ^a^	1350 ± 1.3 ^a^
WD6	8.20 ± 0.6 ^b^	1288 ± 0.4 ^e^	68 ± 0.1 ^d^	43.2 ± 0.3 ^bc^	94 ± 0.5 ^bc^	160 ± 0.5^c^	120 ± 0.4 ^c^	180 ± 0.9 ^cd^	90 ± 0.5	644 ± 0.9 ^d^
WD7	8.60 ± 0.5 ^a^	2300 ± 0.2 ^b^	100 ± 0.5 ^ab^	62.4 ± 0.3 ^ab^	140 ± 0.8 ^ab^	270 ± 0.7^b^	240 ± 0.3 ^ab^	360 ± 0.3 ^b^	190 ± 0.1 ^b^	1150 ± 0.5 ^b^
WD8	8.26 ± 0.7 ^b^	1629 ± 0.6 ^d^	76 ± 0.9 ^cd^	38.4 ± 0.1 ^c^	100 ± 0.9 ^b^	180 ± 0.5 ^c^	157 ± 0.9 ^bc^	220 ± 0.2 ^c^	95 ± 0.6 ^cd^	814 ± 0.8 ^c^
WD9	7.86 ± 0.9 ^e^	550 ± 0.9 ^h^	24 ± 0.8 f.	12.0 ± 0.2 ^e^	50 ± 0.2 f.	70 ± 0.1 ^e^	68 ± 0.1 ^de^	128 ± 0.9 ^e^	30 ± 0.1 ^ef^	275 ± 0.7 f.
WD10	7.83 ± 0.2 ^e^	732 ± 1.0 ^g^	28 ± 0.7 f.	19.2 ± 0.4 ^e^	78 ± 0.3 ^d^	72 ± 0.2 ^e^	67 ± 0.2 ^de^	130 ± 1.1 ^e^	31 ± 0.1 ^ef^	366 ± 0.5 ^ef^
WD11	7.84 ± 0.2 ^e^	1511 ± 1.1 ^d^	40 ± 0.1 ^e^	36 ± 0.3 ^c^	96 ± 0.1 ^bc^	175 ± 0.3 ^c^	150 ± 0.4 ^b^	190 ± 0.9 ^cd^	89 ± 0.0 ^cd^	755 ± 1.0 ^d^
WD12	8.01 ± 0.1 ^d^	654 ± 0.2 ^h^	20 ± 0.5 ^fg^	14.4 ± 0.5 ^e^	70 ± 0.2 ^d^	68 ± 0.4 ^e^	57 ± 0.6 ^e^	115 ± 0.6 f.	24 ± 0.3 f.	326 ± 0.3 ^ef^
WD13	8.03 ± 0.0 ^d^	1351 ± 1.2 ^e^	56 ± 0.3 ^e^	33.6 ± 0.1 ^c^	94 ± 0.1 ^bc^	170 ± 0.2 ^c^	146 ± 0.2 ^bc^	178 ± 0.9 ^d^	71 ± 0.5 ^d^	677 ± 1.0 ^d^
WD14	8.06 ± 0.4 ^d^	1813 ± 1.5 ^c^	80 ± 0.4 ^c^	45.6 ± 0.4 ^bc^	110 ± 0.3 ^b^	185 ± 0.4 ^c^	160 ± 0.9 ^b^	220 ± 0.8 ^c^	93 ± 0.2 ^cd^	907 ± 0.9 ^c^
WD15	7.75 ± 0.6 f.	1421 ± 1.5 ^e^	92 ± 0.3 ^b^	43.2 ± 0.8 ^bc^	98 ± 0.8 ^bc^	175 ± 0.3 ^c^	151 ± 0.1 ^b^	180 ± 0.5 ^d^	73 ± 0.4 ^d^	711 ± 1.1 ^d^
WD16	7.90 ± 0.1 ^e^	388 ± 0.5 ^i^	14.4 ± 0.1 ^g^	45.0 ± 0.9 ^bc^	45 ± 0.4 ^g^	55 ± 0.5 f.	22 ± 0.2 f.	194 ± 0.2 ^cd^	7.9 ± 0.1 ^g^	388 ± 0.8 ^ef^
WD17	7.91 ± 0.7 ^e^	360 ± 0.5 ^i^	32 ± 0.1 f.	16.8 ± 0.1 ^e^	60 ± 0.1 ^e^	52 ± 0.4 f.	48 ± 0.5 ^e^	60 ± 0.1 ^g^	22 ± 0.2 f.	238 ± 0.4 f.

Data presented are means ± SD. Means within columns followed by different letters are significantly (*P* ≤ 0.05) different from each other according to LSD test.

TDS, total dissolved solids.

#### TDS

**Figure 5:**
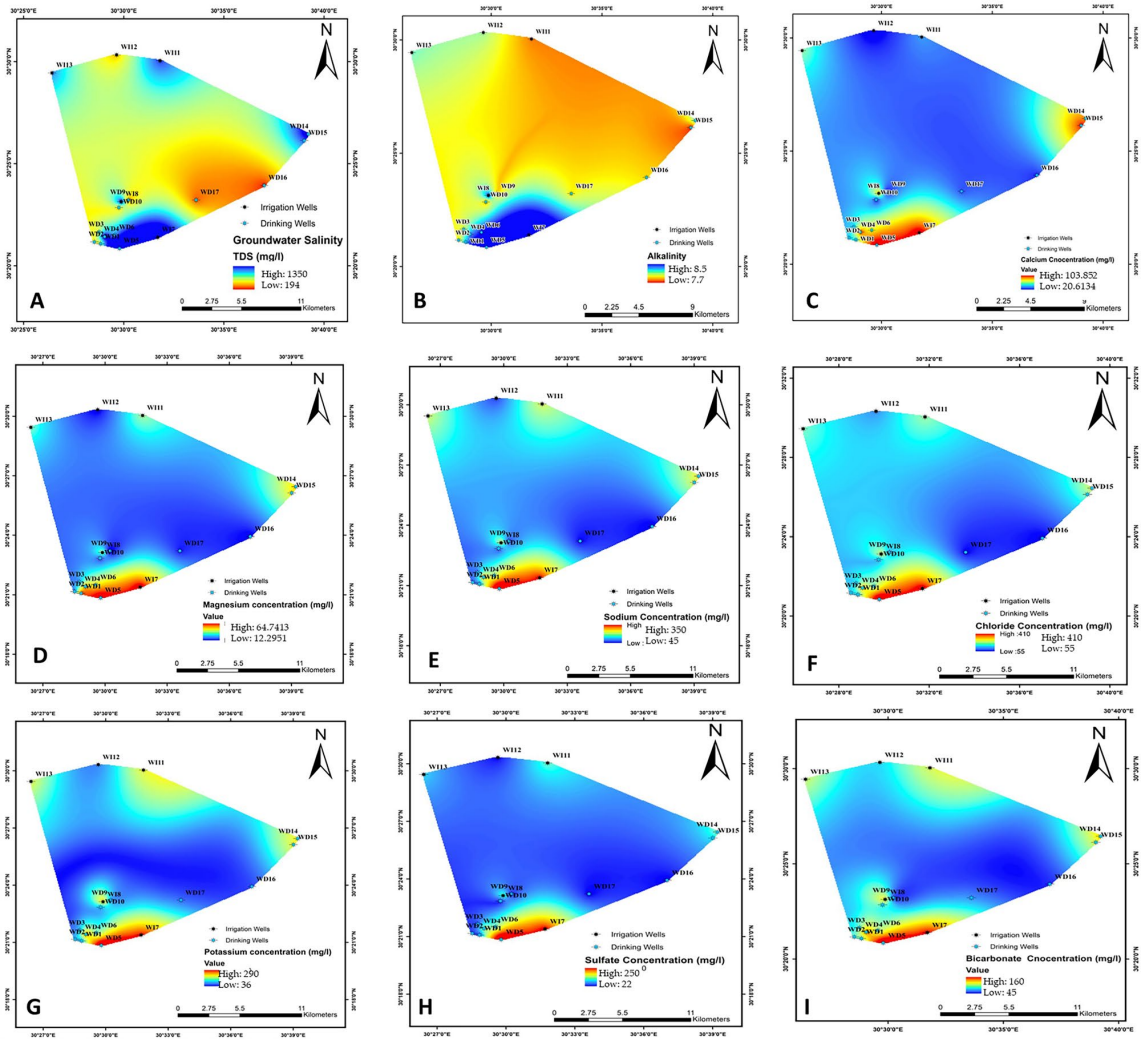
Spatial distribution of physicochemical parameters in groundwater. (**A**) Total dissolved salts (TDS); (**B)** pH; (**C**) calcium (Ca^+2^); (**D**) magnesium (Mg^+2^); (**E**) sodium (Na^+^); and (**F**) chloride (Cl^−^); (**G**) potassium (K^+^); (**H**) sulfate (SO_4_^−2^); and (**I**) bicarbonate (HCO_3_^−^) contents of the examined groundwater.

Salinity distribution in groundwater samples had medium values that varied between 388 ± 0.5 and 1350 ± 1.3 mg L^−1^ (Fig. 5A; Table 2). Water sample #5 (WD5) had the highest salinity, followed by WD7, while WD17 had the lowest. The lowest value (238 mg L^−1^) was observed in the southeast, very close to the eastern boundary of the green belt, while higher values were recorded for most southern wells. The highest salinity value (1350 mg L^−1^) was recorded at the discharge side of the study area. The gradient in salt content was lower on the middle side relative to that on the southern side of the study area. The geographical distribution of water salinity indicated that wells near the wadi and inside the groundwater flow channels had TDS levels surpassing 6 g L^−1^.

#### pH of groundwater

The pH values in the Sadat City region ranged between 7.7 and 8.6 (Fig. 5B; Table 2). WD7 and WD5 samples tend to be alkaline (pH 8.5 and 8.6, respectively). WD15 had a neutral pH, and high pH values were found in the southern part of Sadat City, whereas low values were in the center and northern portions (Fig. 5B; Table 2).

#### Spatial distribution of Ca^+2^

The concentration of Ca^+2^ ions in the study area varied between 14.4 in WD16 and 104 mg L^−1^ in WD5 (Fig. 5C; Table 2). Low Ca^+2^ contents were found in the southeast part of the study area and some places in the northern part, while the samples with high Ca^+2^ contents were found in the southern parts of the study area.

#### Spatial distribution of Mg^+2^

The concentrations of Mg^+2^ ions varied among the tested groundwater samples. Our results showed that the minimum concentration of Mg^+2^ was 12 mg L^−1^ in WD9; whereas the highest was 64 mg L^−1^ in WD7 located in the southern of the study area (Fig. 5D; Table 2).

#### Spatial distribution of Na^+^

As a direct indicator of salinity, Na^+^ ions were the mostly dominant cations in water samples collected in the study area. The highest Na^+^ concentration was 350 mg L^−1^ in WD5; while the lowest was 52 mg L^−1^ in WD17 (Fig. 5E; Table 2). It was found that the northern, eastern, and southern parts of the study area had high concentrations of Na^+^. On the other hand, the southwestern and central parts had lower Na^+^ concentrations; thus, correlating with our results obtained with TDS (Table 2). In general, Na^+^ content in groundwater ranged between 0.05 and 6.9 g L^−1^.

#### Spatial distribution of Cl^−^

Similar to Na^+^ ions, Cl^−^ ions were highly abundant. Even though some water samples, such as WD5 had very high concentrations (410 mg L^−1^), others (WD17) had very low concentrations (60 mg L^−1^) of Cl^−^ (Fig. 5F; Table 2). In comparison, the medium contents varied between 135 and 220 mg L^−1^ in Cl^−^. High values of Cl^−^ ions were observed in the southern part, while low values were found in the eastern part. Medium values of Cl^−^ were found in the southern and western areas of the study (Fig. 5F; Table 2).

#### Spatial distribution of K^+^

WD5 had the highest K^+^ concentrations which is spotted in the southern part of the study area (Fig. 5G; Table 2). However, low K^+^ concentrations were observed in the eastern and southwestern parts, mainly in WD16.

#### Spatial distribution of SO_4_^−2^

In the current study, SO_4_^−2^ concentrations ranged between 7.9 and 250 mg L^−1^ in the measured samples of water (Fig. 5H; Table 2), with the highest in WD5 in the southern parts. The lowest concentration of SO_4_^−2^ was associated with WD16 in the eastern and southwestern parts of the study area.

#### Spatial distribution of HCO_3_^−^

Our results indicated that HCO_3_^−^ were the most common moderate anion. The highest and lowest HCO_3_^−^ concentration was 160 and 45 mg L^−1^ in WD5 and WD16 samples, respectively (Fig. 5I; Table 2). The rest of water samples had medium contents of HCO_3_^−^. The highest concentrations of HCO_3_^−^ were found in the southern part of the study area. On the other hand, the lowest and medium concentrations of HCO_3_^−^ varied between 45 and 100 mg L^−1^ in the northeastern and western parts of the study area, respectively.

#### Microbial load in selected groundwater samples

After conducting a trial on all water samples, high microbial load was found (Table 3). All samples were odorless and did not exhibit turbidity. After two days of incubation, WD14 had the highest bacterial load i.e.*,* 7.5 CFU mg L^−1^. This count was decreased by 40% (4.6 CFU mg L^−1^) in WD17, indicating the lowest microbial load among all samples. Based on our results obtained from the total coliforms, fecal coliforms and fecal streptococci, we did not detect any pathogenic microorganism in the tested water samples.

**Table 3:** The microbial population of groundwater samples in September 2021.

Water sample	Odor	Aspect	Log_10_ CFU mg L^−1^						BGA
			TBC		TC	FC	*E. coli*	FS	
			22 °C/48 h	37 °C/24 h					
WD2	Odorless	Clear	5.3 ± 0.4^b^	4.2 ± 0.2^b^	–	–	–	–	–
WD10	Odorless	Clear	5.0 ± 0.2^b^	3.9 ± 0.1^b^	–	–	–	–	–
WD12	Odorless	Clear	4.8 ± 0.9^c^	3.5 ± 0.3^bc^	–	–	–	–	–
WD14	Odorless	Clear	7.5 ± 0.5^a^	6.7 ± 0.5^a^	–	–	–	–	–
WD17	Odorless	Clear	4.6 ± 0.1^c^	3.3 ± 0.4^c^	–	–	–	–	–

Data presented are means ± SD. Means within columns followed by different letters are significantly *P* ≤ 0.05 different from each other according to LSD test.

*TBC* total bacterial count, *TC* total coliforms, *FC* fecal coliforms, *FS* fecal streptococci, *BGA* blue-green algae,—not detected.

### Types of groundwater in the study area

According to its chemical composition, groundwater can be classified into different types. Here, we used Aquachem (a computer-based program) to classify the groundwater type(s) obtained in the samples of the study area. According to the Rockware Aq. QA program, Na–Cl and Mg–Cl types of groundwater were identified. Na–Cl accounts for 94% of the study area samples; whereas Mg–Cl type represents 6% only (Table 4).

**Table 4:** Types of groundwater samples in the study area in September 2021.

Water type	Total samples (%)	Sample number
Na–Cl	94	WD2, WD3, WD5, WD6, WD7, WD8, WD9, WD10, WD11, WD12, WD13, WD14, WD15, WD16, WD17
Mg–Cl	6	WD1

*WD* water sample.

**Figure 6:**
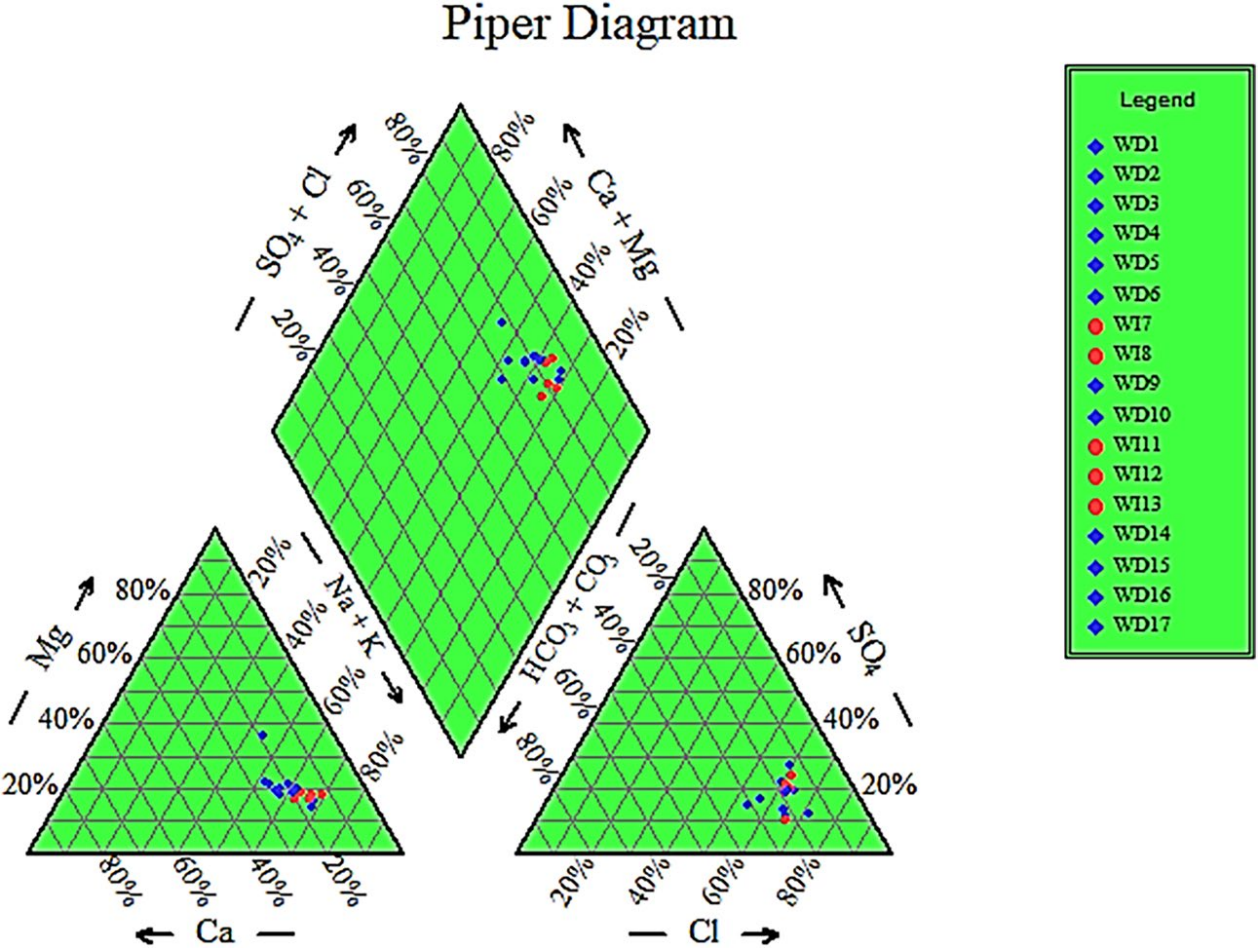
Classification of the groundwater according to Piper diagram.

Based on Piper diagram, Na–Cl groundwater samples possessed brackish water characteristics (Fig. 6). On the other hand, the samples of Mg–Cl type were classified as groundwater with permanent hardness.

### Suitability of groundwater for different purposes

#### Drinking use

The obtained data regarding the groundwater composition are shown in Table 5. According to WHO, seven water samples holding EC values > 1500 μS cm^−1^ were unsuitable for drinking (Table 2). In the study area, three samples of groundwater exceeded the WHO standards' normal limit of Na concentration (200 mg L^−1^), albeit their pH was within the acceptable range. WD5 and WD7 had high values of EC, Ca^+2^, Mg^+2^, CO_3_^−2^, Na^+^, K^+^, Cl^−^, and TDS; while WD1 was high in Ca^+2^ and Mg^+2^, and WD4 was high in K^+^ and Cl^−^ (Table 2). This suggests that all samples can be used as drinking water except for WD5 and WD7.

**Table 5:** The minimum, maximum, and average concentrations of different constituents in September 2021.

Water quality parameter	Groundwater			WHO acceptable limits (2017)	
	Min	Max	Average	drinking water	Irrigation water
EC (μS cm^−1^)	360	2700	1234	1500	2250
Salinity	194	1350	621	1000	2500
Major elements (mg L^−1^)					
Na	45	350	143	200	250
Ca	24	104	57	75	150
Mg	12	64.8	35	50	150
Cl	55	410	183	250	250
SO_4_	22	250	77	250	200
HCO_3_	45	160	92	< 250	300

*EC* electrical conductivity, *Na* sodium, *Ca* calcium, *Mg* magnesium, *Cl* chloride, *SO*_*4*_ sulfate, *HCO*_*3*_ bicarbonate.

#### Irrigation use

EC determines the viability of water samples for irrigation^43^. EC values are shown in Table 6. Five samples were suitable (29.4%), ten had permissible limits (58.8%), and two were doubtful 11.8%. Other criteria, such as salinity, sodium adsorption ratio (SAR) and residual sodium carbonates (RSC), can also be used to investigate the validity of water for irrigation use. In this study, we still classified the water suitability for irrigation used based on the EC values. This can be attributed to the Na concentrations found in the study area that were within acceptable limits (Table 6).

**Table 6:** Irrigation water quality classification depends on electrical conductivity (EC; September 2021).

EC	Water class^106^	Percentage of samples
< 250	Excellent	None
250–750	Good	29.4%
750–2000	Permissible	58.8%
2000–3000	Doubtful	11.8%

### Suitability of groundwater for development

Groundwater development should be subjected to and groundwater conservation and management by the Ministry of irrigation and land reclamation, which is entrusted with recommending the use of this water. According to our analyses, the water is safe and can be consumed by human and for agricultural use. However, groundwater development can be promoted, depending on water demand and plan that is based on comparing the current pumping amount and safe yield by aquifers (Table 7).

**Table 7:** The current rate of groundwater utilization.

Development area (ha)	Current groundwater utilization	Rate of utilization
16,380	Medium	20–40%
5040	Low	< 20%

#### MATLAB simulation

**Figure 7:**
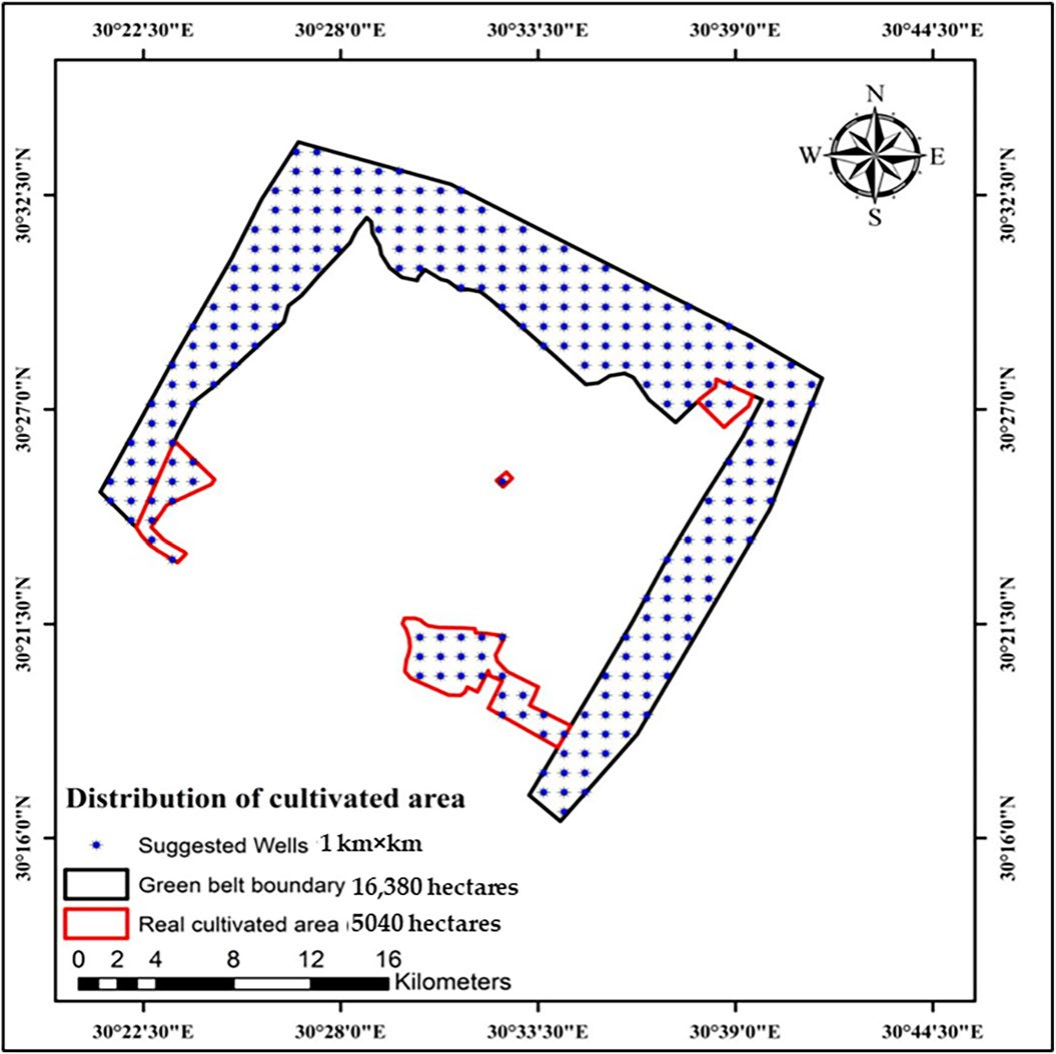
Groups of wells in the development areas.

We generated a new code in the MATLAB program to make an initial simulation of the drawdown values in the wells located in the development area in the Quaternary aquifer by dividing development areas into a group of wells (Fig. 7). The number of development area wells in the 16,380-hectare was 246 wells. The number of development area wells in the 5040-hectare area was 34 wells.

**Figure 8:**
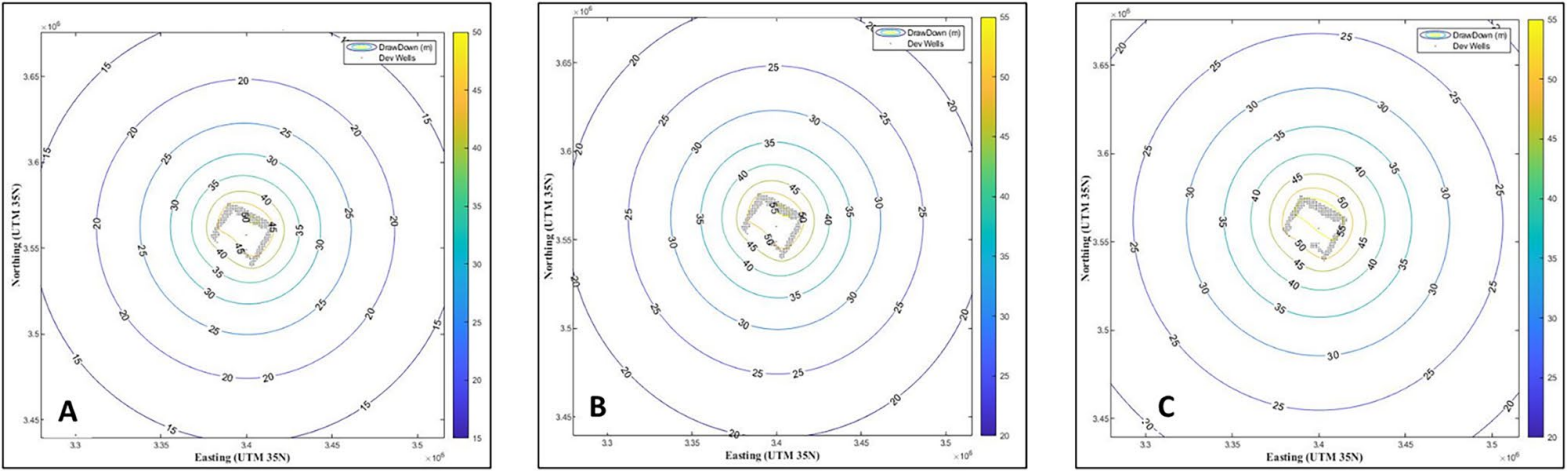
The first simulation model for predicting groundwater development of 16,380 and 5040 ha in the study area. Groundwater drawdown after (**A**) 10; (**B**) 20; and (**C**) 30 years. Numbers on circles indicate the drawdown depth through the years.

**Figure 9:**
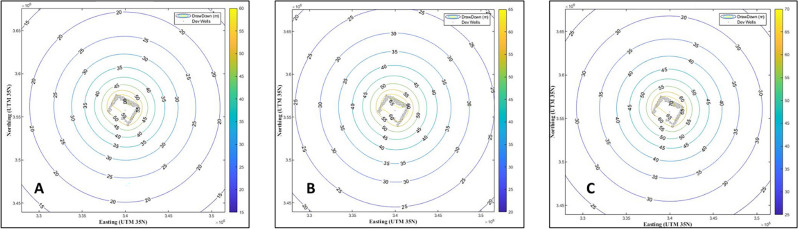
The second simulation model for predicting groundwater development of 16,380 and 5040 ha in the study area. Groundwater drawdown after (**A**) 10; (**B**) 20; and (**C**) 30 years. Numbers on circles indicate the drawdown depth through the year.

The green belt area was estimated to be 16,380 hectares, and the detected wells by GIS in this area were estimated to be 246 (Fig. 7). Only 5040 hectares (31% of the green belt area) were cultivated, containing 34 wells. We also estimated the groundwater quantity in the detected wells for the next 30 years by initiating a new MATLAB code for stimulation (Figs. 8, 9). The development of this area was as the following.

#### Simulation models were divided into two models:

For the first model, the primary transmissivity coefficient (T) = 4000 m^2^ day^−1^, the primary storage coefficient (S) = 0.009028, and the flow rate (Q) = 1500 m^3^ day^−1^. These values refer to the field-estimated values (Fig. 8). The first model, including two development areas of 16,380 and 5040 hectares, with a flow rate of 1500 m^3^ day^−1^ and a T of 4000 m^2^, indicating that most of the development areas are affected by a drawdown of 45 m in the water level in the first ten years, then are affected by a drawdown of 50 m in the water level in the first 20 years of development, and finally are affected by a drawdown of 55 m in the water level in the first 30 years of development. This covers all of the areas of development (Fig. 8).

For the second model, the primary transmissivity coefficient (T) = 4000 m^2^ day^−1^, the primary storage coefficient (S) = 0.009028, and the flow rate (Q) = 1800 m^3^ day^−1^. These values refer to the field-estimated values (Fig. 9). The second model, including two development areas of 16,380 and 5040 hectares, together with a flow rate of 1800 m^3^ day^−1^ and a T of 4000 m^2^ day^−1^, indicated that most of the development areas are affected by a drawdown of 55 m in the water level in the first ten years of development, then are affected by a drawdown of 60 m in the water level in the first 20 years of development, and finally are affected by a drawdown of 70 m in the water level in the first 30 years of development. This covers all of the areas of development (Fig. 9).

## Discussion

Despite being the world's leading groundwater consumer, agriculture faces water scarcity, mainly in arid regions^44^. Irrigation heavily relies on this resource, vital for crop yields and farmland expansion. Unfortunately, environmental issues, human interventions, and natural occurrences jeopardize its quality, thus threatening agricultural sustainability^45^. Urgent action is needed to protect and manage groundwater effectively for long-term agrarian prosperity. Unveiling the hidden world of groundwater chemistry is crucial for ensuring water quality and understanding how seemingly beneficial elements can become silent assassins, leaching away soil vitality and jeopardizing crop productivity^46^.

Groundwater quality is crucial for sustainable agriculture. Indices, such as IWQI, Kelley index (KI), sodium adsorption ratio (SAR), soluble sodium percentage (SSP), potential salinity (PS) and residual sodium carbonate index (RSC), provide valuable insights into the suitability of water for irrigation, helping farmers optimize crop yields and protect soil health. By considering factors, such as salinity, permeability, and chemical composition, indices can inform decisions about crop selection, irrigation practices, and land management strategies^47^. Researchers are delving into the secrets of groundwater suitability for agriculture, from arid landscapes to lush farmlands. Tools, such as IWQI and GIS, are invaluable. By analyzing vast amounts of data on water chemistry and mapping it onto interactive platforms, these technologies reveal hidden patterns and create "quality zones" for irrigation^48,49^.

Sustainable farming needs fresh perspectives on groundwater management. Predicting water quality using artificial intelligence empowers farmers to make informed decisions about irrigation practices, crop selection, and long-term land use^50,51^. By illuminating the hidden corners of water quality, Machine learning (ML) algorithms has the potential to revolutionize how we protect this precious resource. Farmers can optimize irrigation based on artificial intelligence-powered forecasts or cities proactively treating contaminated water before it becomes a crisis. This is the future that artificial intelligence promises, a future where data become the key to unlocking sustainable water management for generations to come^52^.

Many investigations were carried out within the designated research zone to monitor water levels, measure hydraulic parameters, evaluate potable water health hazards, and analyze temporal soil quality degradation^53,54^. As a result, routine monitoring and evaluation of the irrigation water quality in the region of Sadat City is necessary to determine how anthropogenic activities, rapid water level withdrawal, and geological composition may impact the irrigation water quality, affecting soil quality and crop production^55^.

According to the report provided by the Food and Agriculture Organization (FAO) of the United Nations, the highest EC value was 2610 μS cm^−1^, which is below the limits of 3000 μS cm^−1^ in El Kharga Oasis, New Valley Governorate, Egypt^56^. The pH values ranged between 6.1-8.1, is also within the acceptable limits for water use in irrigation^57^. Our results agreed with other studies^58^, which found that pH varied between 7.5-9.9. In the current study area, pH variations may have occurred due to the minerals, pollutants, soil or bedrock composition, and other contaminants interacting with water^59^.

Except of two samples, the observed TDS in the current study was < 1000 mg L^−1^, which was also within the standard limits^60^. Consistent with that, Lahjouj et al.^61^ have reported that TDS values are 492.73 and 526.61 mg L^−1^ in spring and autumn, respectively, in Sais Basin, Morocco. In Essaouira, Morocco, TDS was low (390–874 mg L^−1^) in Krimat aquifer, while other samples show high values (958–2520 mg L^−1^)^62^. The majority of the groundwater in Talukas of Tharparkar, Southern Sindh, Pakistan have TDS values of 800–11,000 mg L^−1^^63^. Thus, these wells have been reported to cause major health problems in the area. Qureshi et al.^64^ also assessed the groundwater quality from samples at different locations of Tandojam, Pakistan, and found that TDS and other physicochemical parameters exceeded the WHO guidelines in most locations, but were acceptable in few *i.e.,* Sindh Agriculture University. In alignment with that, the water quality in the Garibabad colony, Shahi Bazar, and Pakistani Chowk was found to be salty and has high levels of hardness; therefore, rendering it unsatisfactory^64^.

Na^+^ concentration, ranging 4–460 mg L^−1^, was within the acceptable value for the examined groundwater. In Krimat aquifer, Morocco, Na^+^ concentrations vary between 10.4 and 303.3 mg L^−1^, averaging 134.2 mg L^−1^^62^. The Cl^−^ (175.53 mg L^−1^), and SO_4_^2−^ (143.47 mg L^−1^) were the most abundant anions in the groundwater. Thus, their overall concentrations were permissible for irrigation water, where Cl^−^concentrations ranging 99.4–1178.6 mg L^−1^, and an average of 427.5 mg L^−1^ in Krimat aquifer. The high Cl^−^ concentration can be attributed to the dissolution of halite^65^.

Although K^+^ concentrations in all locations exceeded the allowable irrigation water standards by a minute (3.50–53.00 mg L^−1^), this was consistent with the study of El Mountassir et al.^62^ who reported a range of 0.3–36.5 mg L^−1^. The K^+^ concentration is greatly dependent on the sediment type in the aquifer media, ranging from 22 to 290 mg L^−1^. Similar values have also been reported in other studies^58,66^. The considerable K^+^ content may be attributed to the silicate minerals, orthoclase, microcline, hornblende, muscovite, and biotite in igneous and metamorphic rocks and evaporate deposits. SO_4_^−^ and gypsum release large amounts of K^+^ into groundwater^67^. Pandey et al.^66^ determined SO_4_^−^ values between 59.90–294.30 mg L^−1^^68^. The variations in SO_4_^−^ in groundwater may be due to the presence of evaporite rocks with high SO_4_^−^ concentrations. In addition, it might be attributed to the evaporite rocks with high SO_4_^−^ concentrations and contaminant fertilizers^68,69^.

HCO_3_^−^ concentrations were acceptable in all samples to be used for irrigation with a maximum of 300 mg L^−1^. This may explain the recharge effects of the intensive irrigation practices and irrigation canal systems along the boundary of the green belt, where salt dissolves and hence the salt content increases in the groundwater. Our results are consistent with other reports. For example, Lahjouj et al.^61^ have reported HCO_3_^−^ content ranging between 280 and 308 mg L^−1^ in Sais Basin, Morocco. Pandey et al.^66^ have also measured HCO_3_^−^ in about the same range (268 to 516 mg L^−1^) in Allahabad smart city, India. The higher HCO_3_^−^ values in the study area's southern parts may be due to the dissolution of carbonate materials in the water-bearing formations. Additionally, the granite gneiss contains orthoclase feldspar and biotite minerals, which after weathering, yield HCO_3_^−^- and chloride-rich groundwater^70^.

Ca^+2^ ion variations may occur in the study area because of the presence of Ca in geologic materials, such as gypsum, calcite, and dolomite in the subsurface layers^71^. The average concentration is present in the middle of the sample map, and that might be attributed to cation exchange (Fig. 5D; Table 2). Ca^+2^ concentration of in all samples met the water irrigation standard (8–180 mg L^−1^). Only 1.42% of the samples had higher Mg^+2^ concentrations (above the acceptable levels of irrigation water), while the rest had a mean concentration of 21.90 mg L^−1^. In Tunisia, Mg content in groundwater ranges between 17.3 and 242 mg L^−1^^58^; whereas in Morocco, Ca content ranges between 38 and 54 mg L^−1^^61^. This may be attributed to the lithological effect from the dissolution of carbonate rocks in the catchment area and the Mg-rich minerals in the cement materials of the Quaternary aquifer system in the study area^72^.

In addition, the group "Shallow well (3)" predominates Na^+^ and Cl^-^ in groundwater samples; these samples are rich in HCO_3_^−^, which may release Na^+^ ions into the solution through an exchange reaction^73^. The high concentrations of Na^+^ cations in the study area may be attributed to the high solubility of evaporite rocks in the fluviomarine deposits. In addition, clay layers with fluvial sand often yield water with a relatively high Na^+^ content^74^. An excess in HCO_3_^−^ may cause the release of Cl^−^ into the solution by exchange reactions within the exchange sites^75^. The increased Cl^−^ concentrations found may be attributed to a probable presence of evaporite rocks in the lithological units in the study area.

The Chadha diagram further confirmed the primary dominating geochemical processes controlling water chemistry^76^. The majority of samples (71.43%) were situated in the Na–Cl type region. This suggests that the dissolving of halite minerals is a substantial determinant in groundwater chemistry. Around 14.28% of the water samples were found in the reverse ion exchange zone (Ca–Mg–Cl/SO_4_), whereas 10.72% were in the base ion exchange zone (Na-HCO_3_). The remaining 3.57% of the samples were in the recharge water zone (Ca–Mg–HCO_3_). Notably, groundwater's suitability for irrigation water uses, and its evolution in quality is contingent upon geochemical processes and the control mechanism. Consistent with El Mountassir et al.^62^, the Ca–Mg–HCO_3_ and Na-HCO_3_ water types identified in the chemical analysis of the groundwater samples correspond to the meteoric/initial water stages in the recharge locations. The facies characterizing the study area was a combination of Ca–SO_4_ and mixed Ca–Mg–Cl^62^. Based on the bivariate diagrams of major ions, the hydrochemical approach indicates that the origins of groundwater mineralization are the result of evaporite dissolution, cation-exchange reactions, and evaporation processes.

The Na–Cl groundwater type is present near the boundary of the study area's green belt and the central cultivated area. This may be attributed to the external contamination caused by the sewage leakage and the agricultural drainage to groundwater formations, in addition to the leaching of salts from the evaporite sediments or the adsorption process of sodium on clay, which increases the sodium concentration^66^.

The Mg–Cl type exists in one isolated location in the south-central zone of the study area. This may be attributed to the dissolution of evaporite rocks or the magnesium-rich minerals of the Quaternary aquifer system in the study area, causing cation exchange^66^. Ca^+2^ and Mg^+2^ ion concentrations in the groundwater samples did not exceed the international standards of the WHO^77^. Concerning K^+^, all water samples exceeded the limit of the WHO and other guidelines standards **(**Table 1)^78–80^. In contrast, the Ca–Mg–Cl/SO_4_ water type, particularly in the northern and central regions of the study area, reflected the intermediate water stages of evolution. Furthermore, most samples belonged to the Na–Cl water type, which signifies the final phase of geochemical transformation in the discharge regions, particularly in the southern sections of the research area where groundwater flow is observed. By utilizing geochemical modeling to ascertain the mineral saturation state, these results may validate prior research in the topic^81^. Utilizing the link between the EC and Na^+^/Cl^−^ ratio, the influence of the dissolution and evaporation processes in the research region could be explained^82^.

In the unsaturated zone, halite dissolution influences are typical of arid and semi-arid regions with annual precipitation averages below 600 mm^83^. In the samples located beneath the 1:1 line graph, an increase in Cl^−^ concentration could indicate an extra source of Cl^−^ ions. In contrast, a decrease in Na^+^ concentration could be attributed to the elimination Na^+^ ions from the groundwater. Human activities such as waste disposal and excess irrigation water from agricultural land may contribute to elevated Cl^−^ levels^84^ or Cl^−^ deposition in the atmosphere^85^. Certain samples exhibited Na^+^/Cl^−^ ratios exceeding 1, which may suggest the occurrence of silicate weathering or ion exchange^86^.

In most water samples, Na^+^ and K^+^ predominated over Ca^+2^ and Mg^+2^, indicating that Ca^+2^ and Mg^+2^ ions were replaced by Na^+^ and K^+^ ions via ion exchange and silicate weathering^87^. The reverse ion exchange process, showed that only a few groundwater samples surpassed the 1:1 line, represented by the sum of Ca^+^^2^ and Mg^+^^2^ ions against the sum of HCO_3_^−^ and SO_4_^–2^. As a result of dolomite, calcite, and gypsum degradation, the samples surpassed the 1:1 line. The proportional increase in SO_4_^2−^ and HCO_3_^−^ ions relative to Ca^+2^ and Mg^+2^ ions can be attributed to silicate weathering^88^. This is evident in the Na/Cl ratio, which indicates that the water was more enriched in Na^+^ than Cl^−^. The proportion of Ca^+2^ and Mg^+2^ to HCO3^−^ could be utilized to determine the source of calcium and magnesium in the samples. If the ratio is near 0.5, carbonate and silicate minerals weathering produced the Ca^+2^ and Mg^+2^^89^.

Because agricultural practices, soil types, and water quality all influence the most suitable irrigation methodologies^90^, IWQI, SAR, SSP, KI, PS, and RSC, were utilized in this study to monitor the water quality suitability for agriculture. These methodologies draw attention to the potential for salinization of the soil and the detrimental effects of irrigation on the well-being of plants and soil. For irrigation purposes, groundwater is evaluated by utilizing IWQI and either individual chemical indices^91^ or a combination of multiple indices^92^. While considering groundwater for irrigation using individual parameters is crucial, combined indices offer decision-makers more valuable information. Utilizing five hazard categories, the safety of the groundwater for irrigation purposes was assessed^60^. The "SAR parameter" in irrigation water pertains to the capacity of the soil matrix to release Ca^+2^ and Mg^+2^ ions while absorbing Na^+^ ions at the sites of ion exchange. This process results in the dispersion of soil particles and a decrease in infiltration capacities^93^. Although applying highly saline water to irrigate crops can enhance infiltration and improve soil structure, it places plants under increased water stress. To extract water from the soil, plants, and crops are compelled to utilize more energy, known as the water stress condition. The United States salinity laboratory (USSL) diagram classifies and illustrates the correlation between SAR and EC^94^.

The precipitation of Ca^+2^ and Mg^+2^ in the form of carbonate minerals may result in an elevation of Na^+^ concentrations and, consequently, SAR values^95^. A high value of the RSC can lead to a degradation of the physical properties of the soil through the dissociation of organic matter. This results in a desiccated, black discoloration on the soil surface^96^. The RSC was calculated to forecast the likelihood of Ca^+2^ and Mg^+2^ precipitating on soil surface particles and their subsequent removal from the soil solution. Reports indicated that desiccated and semi-arid regions have elevated RSC concentrations in groundwater, leading to soil salinization and sodification^97^. Three distinct kinds of groundwater were identified using RSC^97^. Consistent with other study^98^, the land resources and soil classification demonstrated that the soils were categorized as mediocre, poor, or very poor based on their salinity, alkalinity, and texture.

An investigation carried out in Ghana concerning irrigation water^99^ revealed elevated levels of *E. coli*, compared to the findings of our study. The mean population of *E. coli*, as determined by sampling locations, exceed the suggested threshold of 2.3 log_10_ CFU 100 mL^−1^. This result indicates that irrigation water is extremely contaminated with microorganisms. Contrary to the findings of Khan et al.^47^, the population of sulfite-reducing clostridia and fecal enterococci were greater and lower, respectively. The potential origins of fecal pollution in irrigation water are manifold, resulted from municipal sewage and agricultural practices. In light of the exorbitant expenses associated with industrial fertilizers, market gardeners employ substantial poultry and cattle droppings as soil amendments^47^. These methods will probably result in the contamination of irrigation water, particularly that sourced from wells. Undoubtedly, these wells are situated in market horticulture areas and are shallow and exposed. Wastewater is frequently released into the environment without undergoing any form of remediation. Microorganism-laden water accumulates in receptacles such as shallows and dams, explaining the high levels of contamination observed in these two locations throughout this investigation. Shallow water, as opposed to dam water, is stagnant. This attribute might account for the greater level of pollution observed in shallow water as opposed to dam water. Keesari et al.^100^ found that the total bacterial count (TBC) in 29 groundwater samples from various aquifers was 1.8 × 10^3^ CFU mg L^−1^, belonging to 5–10 different microbial strains. The TBC distribution in groundwater depends on the study area's human and agricultural activities and chemical properties.

The absence of fecal pollution is a critical indication of water quality. Generally, the groundwater is contaminated by pathogens from infested human feces that spread from the soil to water. There are several kinds of microorganisms in groundwater, including viruses, bacteria, and parasites. Common human bacterial gut diseases, including salmonellosis, cholera, and shigellosis, are spread predominantly through contaminated water from sick individuals' feces^7,101^. The danger of water-borne infections increases as fecal pollution rises^102^.

Air, soil, and fungal spores have the potential to pollute irrigation water^103^. The obtained mold isolates were classified within the genus *Aspergillus*. This result supports the findings of Akinde et al.^104^ who investigated the relationship between irrigation water and fresh vegetables in southeastern Nigeria. Furthermore, the authors demonstrated that these isolates were capable of producing mycotoxins. There is a potential for vegetable products to be contaminated by irrigation water. Numerous investigations^104,105^ have previously established the correlation between irrigation water and vegetable contamination. The environmental preferences of mesophilic aerobic microorganisms, sulfite-reducing clostridia, yeasts, and total coliforms are found to be similar to those of molds and fecal enterococci, as indicated by the results of pH, temperature, conductivity, and TDS. The significant correlation between pH and mold demonstrates the acidophilic character of most of these microorganisms. The potential origins of mesophilic aerobic microorganisms, sulfite-reducing clostridia, yeasts, total coliforms, and *E. coli* contamination are consistent with ammonium, as indicated by the robust correlation observed among these parameters.

Detecting issues at an early stage will facilitate the implementation of preventative measures for this critical resource. The output parameter is the IWQI, which is predicted by artificial intelligence algorithms using the following input parameters: water EC, Na concentration, SAR, Cl and HCO_3_ concentrations. These parameters were computed by analyzing 166 samples gathered from an arid desert. Recently, a study has reported that producers in arid regions can increase agricultural output by improving irrigation water quality management, and policymakers and stakeholders can make informed decisions regarding water resource management^53^. IWQI serves as a valuable instrument for stakeholders and producers to evaluate the appropriateness of groundwater for agricultural purposes, specifically concerning irrigation. By comparing the anticipated IWQI with the prescribed benchmarks, decision-makers can ascertain whether the groundwater is appropriate for irrigation or if further treatment protocols are required. The data mentioned above is crucial to maximize agricultural output, mitigating the adverse effects of substandard water quality on crops, and establishing sustainable practices for managing water resources.

Furthermore, concerning potable water, the anticipated IWQI facilitates the assessment of the groundwater's appropriateness for human ingestion. Using the drinking water standards by WHO, decision-makers can evaluate the potential health hazards linked to groundwater consumption by comparing the computed IWQI. This information is of the utmost importance in guaranteeing that communities have access to potable water, as it facilitates the detection of situations that require alternative water sources or suitable treatment methods. Our research equips stakeholders and decision-makers with a dependable and effective instrument for assessing groundwater quality. This enables them to make knowledgeable judgments concerning water resources management and protects the health and welfare of communities that depend on groundwater for drinking and irrigation.

## Conclusion and future prospective

The current study utilized physicochemical parameters, IWQI, GIS tools, and a new MATLAB code to identify groundwater hydrogeochemical classes and their governing processes to assess the groundwater's suitability for agricultural purposes in Sadat City, Menoufia Governorate, Egypt. Based on the physicochemical data collected, the groundwater resources consisted of Na–Cl and Mg–Cl. To forecast the IWQIs, two simulation models were constructed using these physicochemical parameters. The performance evaluation indicates that the MATLAB modeling achieved satisfactory accuracy in simulating the IWQI during the learning and validation phases. The precise performance of the proposed models suggests that they may be viable for predicting IWQI. Hence, the integration of physicochemical parameters, IWQIs, GIS, and the viability of ML models can effectively facilitate the application of groundwater for irrigation objectives. Our results will enhance the knowledge of the precise and coordinated administration of water resources in Sadat City. This study provides a dependable technology for contingency plans involving water resources risks. Consequently, in the future, it will be advantageous to regulate the environmental protection of the water environment.

Based on the hydrochemical and physicochemical properties of the groundwater samples, we can conclude that all groundwater samples can be used as drinking water after some treatments, except for WD5 and WD7 samples. Based on MATLAB simulation models, there is a drawdown in the groundwater quantity in the next 10–30 years from 45 to 70 m. Therefore, the continuous monitoring of groundwater levels in the study area should be conducted via a monitoring network. The groundwater quality should be monitored by conducting physical, chemical, and microbiological analyses to ensure the quality of this water for various uses, especially for drinking wells in the study area. In a future study, we will design a new MATLAB code to predict the quantity and quality of groundwater in the study area, including the contamination sources. The approach proposed in this study can be subjected to additional research to enhance its precision for groundwater across diverse circumstances. Moreover, it empowers decision-makers to integrate multiple technologies in the context of water quality planning and management.

### Supplementary Information

Supplementary Information.

## Data Availability

The data presented in this study are available upon request from the corresponding author.
