# Supplementary material for: Integrated management of groundwater quantity, physicochemical properties, and microbial quality in West Nile delta using a new MATLAB code and geographic information system mapping
Source: Sci Rep. 2024 Apr 2;14:7762. doi: 10.1038/s41598-024-57036-8 (PMC10987591; doi:10.1038/s41598-024-57036-8)
Supplement: Supplementary file 1 — Supplementary Information. [file 41598_2024_57036_MOESM1_ESM.pdf]

Supplementary Materials

Integrated Management of Groundwater Quantity, Physicochemical Properties, and Microbial Quality Using A New MATLAB Code and Geographic Information System Mapping

Mohamed Shehata, Samir M. Zaid, Soha T. Al-Goul, Ashwag Shami, Khalid M. Al Syaad, Ahmed Ezzat Ahmed, Yasser S. Mostafa, Diana A. Al-Quwaie, Mada F. Ashkan, Fatimah S. Alqahtani, Yusuf A. Hassan, Taha F. Taha, Khaled A. El-Tarabily, Synan F. AbuQamar*

*** Correspondence:**

Prof. Synan AbuQamar: sabuqamar@uaeu.ac.ae

Table S1. Main composition for layers sequence of wells in study area.

Depth (m)		Description	Lithological photo
**From**	**To**		
**0**	**60**	**Rock fragments:** Medium to fine sand, Qz, pale yellow, transparent, off white, colorless, hard with clay, in part brown, soft, flacky.	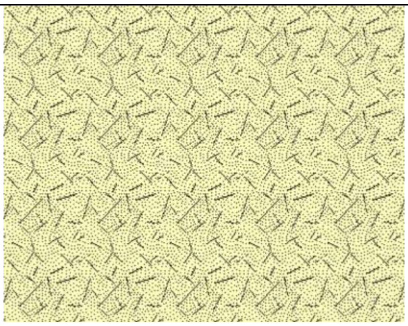
**60**	**160**	**Sand:** Fine to medium, Qz, colorless, light gray, off white, hard.	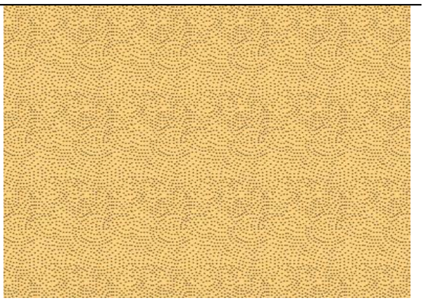
**160**	**200**	**Sand:** Fine to medium, Qz, colorless, light gray, off white, hard with shale, hard, gray, sticky	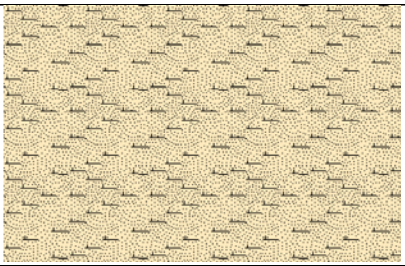
